# Sleep quality of adult recreational cannabis users: a systematic literature review

**DOI:** 10.47626/2237-6089-2024-0802

**Published:** 2025-05-26

**Authors:** Nathália Lima Costa, Wennyo Camilo da Silva e Silva, Marianne Lucena da Silva, Katiane da Costa Cunha

**Affiliations:** 1 Universidade do Estado do Pará Marabá PA Brazil Universidade do Estado do Pará, Marabá, PA, Brazil.; 2 Universidade do Estado do Pará Marabá PA Brazil Universidade do Estado do Pará, Marabá, PA, Brazil.; 3 Universidade de Brasília Brasília DF Brazil Universidade de Brasília (UnB), Brasília, DF, Brazil.; 4 Universidade do Estado do Pará Marabá PA Brazil Universidade do Estado do Pará, Marabá, PA, Brazil.

**Keywords:** Sleep, cannabis, recreational marijuana use, quality of life

## Abstract

**Objective:**

Human sleep is fundamental for the proper occurrence of organic functions. Hence, lack of sleep can impair cognitive function, resulting in emotional problems, memory changes, and disease onset. However, it is worth noting that sleep is influenced by outside factors, such as drug use. In this sense, the aim of this review is to analyze studies that researched the influence of recreational marijuana on the sleep quality of adults.

**Methods:**

High-sensitivity searches were conducted on databases (Biblioteca Virtual em Saúde [BVS], MEDLINE via PubMed, Cochrane, Embase, Web of Science, and Scopus) using descriptors related to marijuana and sleep habits.

**Results:**

Eighteen studies from four countries were included with a total sample size of 29,858 participants. It was found that marijuana affects sleep characteristics – such as latency and duration – with these changes being more evident in users who make greater use of marijuana and in those with early onset of marijuana use.

**Conclusion:**

It was observed that most articles demonstrated a detrimental effect of recreational cannabis use on the quality of sleep in adults.

## Introduction

Most organisms possess circadian rhythms, as a decrease in wakefulness during some part of the 24-hour day is essential. Human sleep is divided into two main phases, rapid eye movement sleep (REM) and non-rapid eye movement sleep (NREM). During these phases, physiological changes such as muscle relaxation and changes in respiratory rate are common. These oscillations are necessary to attains each of the sleep phases, provoking reductions in the state of consciousness and sensory and motor responses, so that sleep achieves its restorative function.^1^

From childhood, maintenance of an ideal circadian rhythm is important, as inadequate sleep can promote morphological changes in the hippocampus, amygdala, and prefrontal cortex.^2^ Insufficient restorative sleep can harm human health. It is known that lack of sleep can increase the risk of obesity, thus contributing to emergence of chronic non-communicable diseases – diabetes mellitus and arterial hypertension – and cardiovascular diseases, such as acute myocardial infarction, in addition to being associated with stress and depression.^3^

An adequate sleep pattern is influenced by external factors. Psychological health, infections, use of medications, and even lifestyle habits can negatively affect sleep. In fact, these different scenarios can prevent individuals from reaching deep sleep, causing nighttime awakening, or they can cause hypersomnia, resulting in sleep disorders, which impair the patient's studies, work, and mood.^4^

One of the main lifestyle habits that damages sleep quality is use of drugs, which is considered the main risk factor for the onset of sleep disorders. One such drug is marijuana. It is believed that marijuana derivatives can be useful in the treatment of insomnia, being considered research targets in patients who suffer from sleeping difficulties.^5^ However, long-term marijuana use can be harmful to the quality of sleep, potentially reducing users’ total sleep time.^6^

Data from the United Nations Office on Drugs and Crime (UNODC) reveal that marijuana is the most widely used drug in the world, with an estimated 219 million users.^7^ It should be noted that the number of people who consider marijuana as harmful has reduced, while the potency of cannabis has increased by up to four times as legalization has advanced during the 21st century, causing damage to the health of its users that is only noticeable after years of use.^8^ Within this context, this review aims to analyze studies that address the effects of recreational marijuana on the sleep of adult users.

## Methods

This systematic review was based on quantitative data published in articles and follows the recommendations and criteria described in the Preferred Report Items for Systematic Reviews and Meta-Analysis (PRISMA)^9^ guideline and the Cochrane Handbook.^10^

### Search strategy

Potential studies were identified with a comprehensive search strategy. The systematic review was carried out using the following databases: Virtual Health Library (Biblioteca Virtual em Saúde [BVS]), MEDLINE via PubMed, Cochrane, Embase, Web of Science, and Scopus. There were no language restrictions. The search strategy involved combinations of selected keywords from the Medical Subject Headings (MeSH) and the Descritores em Ciência da Saúde (DeCS) libraries:

– BVS – (cannabis OR marihuana) AND (sono OR sueño OR sleep OR hábito de dormir OR hábitos de dormir OR hábitos do sono OR qualidade do sono OR avaliação do sono OR escala do sono OR questionário do sono)
– MEDLINE via PubMed – ("Cannabis"[MeSH] OR Cannabi OR "Hemp Plant" OR "Hemp Plants" OR Marihuana OR Marijuana OR "Cannabis indica" OR "Cannabis sativa" OR Hemp OR Hemps OR Hashish OR Hashishs OR Bhang OR Bhangs OR Ganja OR Ganjas) AND ("Sleep"[MeSH] OR "Sleeping Habits" OR "Sleep Habits" OR "Sleep Habit" OR "Sleeping Habit" OR "Sleepiness Scale" OR "Sleep Scale" OR "Sleep Questionnaire")
– COCHRANE – (Cannabis OR Cannabi OR "Hemp Plant" OR "Hemp Plants" OR Marihuana OR Marijuana OR "Cannabis indica" OR "Cannabis sativa" OR Hemp OR Hemps OR Hashish OR Hashishs OR Bhang OR Bhangs OR Ganja OR Ganjas):ti, ab, kw AND (Sleep OR "Sleeping Habits" OR "Sleep Habits" OR "Sleep Habit" OR "Sleeping Habit" OR "Sleepiness Scale" OR "Sleep Scale" OR "Sleep Questionnaire"):ti, ab, kw"
– Embase – ‘sleep’/exp OR sleeping AND ‘cannabis’/exp OR bhang OR "cannabis alkaloid" OR "cannabis constituent" OR "cannabis extract" OR "cannabis herba" OR "cannabis leaf" OR "Cannabis sativa extract" OR "Cannabis sativa leaf" OR "Cannabis sativa resin" OR cannador OR charas OR ganja OR ganjah OR hashish OR "hashish oil" OR "hemp extract" OR "herba cannabis" OR "Indian bhang" OR "Indian ganja" OR marihuana OR marijuana OR "mexican marihuana"
– Web of Science – (ALL=(Cannabis OR Cannabi OR "Hemp Plant" OR "Hemp Plants" OR Marihuana OR Marijuana OR "Cannabis indica" OR "Cannabis sativa" OR Hemp OR Hemps OR Hashish OR Hashishs OR Bhang OR Bhangs OR Ganja OR Ganjas)) AND ALL=(Sleep OR "Sleeping Habits" OR "Sleep Habits" OR "Sleep Habit" OR "Sleeping Habit" OR "Sleepiness Scale" OR "Sleep Scale" OR "Sleep Questionnaire")
– Scopus – (TITLE-ABS-KEY (cannabis OR cannabi OR "Hemp Plant" OR "Hemp Plants" OR marihuana OR marijuana OR "Cannabis indica" OR "Cannabis sativa" OR hemp OR hemps OR hashish OR hashishs OR bhang OR bhangs OR ganja OR ganjas) AND TITLE-ABS-KEY (sleep OR "Sleeping Habits" OR "Sleep Habits" OR "Sleep Habit" OR "Sleeping Habit" OR "Sleepiness Scale" OR "Sleep Scale" OR "Sleep Questionnaire"))

### Inclusion criteria

Eligibility criteria were established from the PVO model (Participants, Variables, Outcomes), which generated the research question: Does recreational marijuana interfere with the sleep quality of its users? The following inclusion criteria were adopted for study selection: observational studies carried out with humans over 18 years of age that evaluated sleep quality or sleep-related problems in individuals who used recreational marijuana.

### Exclusion criteria

Studies with the following characteristics were excluded: non-observational studies, studies that analyzed sleep without relating it to the use of marijuana, studies of medicinal marijuana users, those in which the methodology was not clearly written, as well as books, letters to the editor, and case reports.

### Data extraction

**Figure 1:**
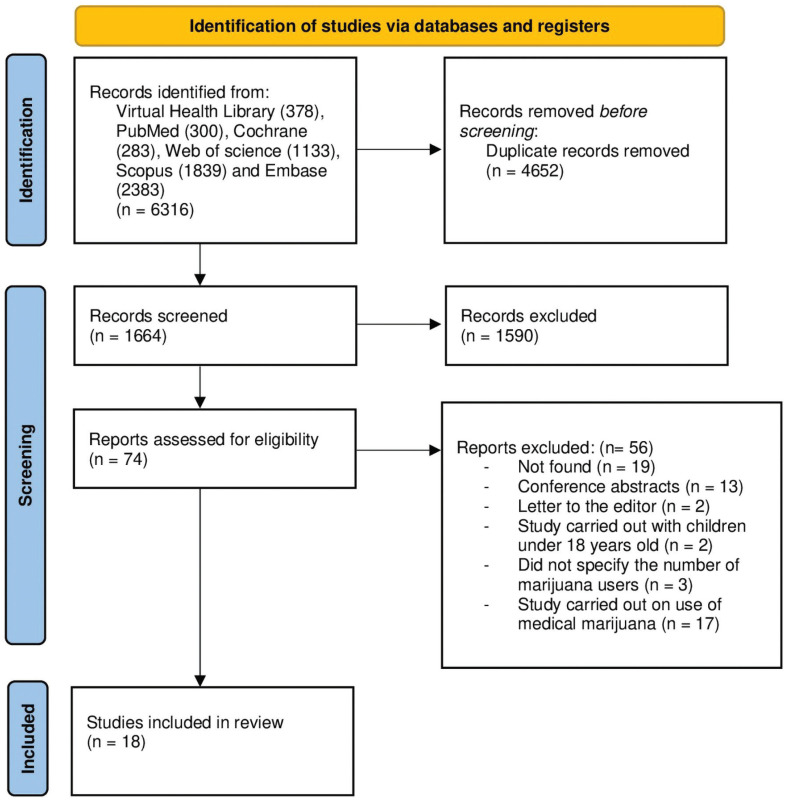
Preferred Report Items for Systematic Reviews and Meta-Analysis (PRISMA) flow diagram of studies that assessed sleep quality.

The material obtained through the database searches was exported to the Rayyan^®^ and Mendeley^®^ platforms and summarized in a PRISMA diagram (Figure 1). The first screenings, by title and abstract, were carried out by three independent researchers (WCSS, NLC, and MLS), selecting possible articles to be included in the final compilation. In cases in which there were disagreements, discrepancies were resolved by a fourth independent researcher (KCC). For data extraction, the three independent researchers (WCSS, NLC, and ML) used Microsoft Excel^®^ spreadsheets to record the following: study data (authors, title, and publication year) and methodological information (design, sample size, sleep quality variables or aspects, and instruments used for assessment).

### Quality analysis

Two authors (WCSS and NLC) performed independent assessments of the quality of the studies using two scales, the Newcastle-Ottawa Scale (NOS) and the Joana Briggs Institute (JBI) scale. Table 1 shows the results of the NOS assessment. This scale uses star ratings (0-10) to assess studies in three domains: selection, comparability, and results. Higher scores indicate higher quality studies. The risk of bias, also assessed by both authors (WCSS and NLC), was rated using the JBI checklist. This checklist contains nine questions divided into three domains: participants (questions 1, 2, 4, and 9), measurement of results (6 and 7), and statistics (3, 5, and 8).

**Table 1:** Methodological quality of the studies according to the Newcastle-Ottawa Scale (NOS)

Author	Representativeness of the sample	Comparability	Outcome	Total score
Diep et al.^11^	5	1	2	8
Drazdowski et al.^12^	4	1	2	7
McPherson et al.^13^	5	1	2	8
Skobic et al.^14^	3	1	2	6
Wheeler et al.^15^	5	2	2	9
Winiger et al.^16^	4	1	2	7
Winiger et al.^17^	5	2	2	9
Winiger et al.^18^	3	1	2	7
Winiger et al.^19^	4	2	2	8
Sznitman et al.^20^	3	1	2	7
Altman et al.^21^	4	2	2	8
Bolla et al.^22^	4	1	3	8
Fisk et al.^23^	4	1	2	7
Karacan et al.^24^	3	1	3	7
Lim et al.^25^	3	1	2	6
Ly et al.^26^	4	1	2	7
Maple et al.^27^	4	1	2	7
Ogeil et al.^28^	4	2	2	8

## Results

### Selection and evaluation of studies

The initial searches returned 6,316 studies found in the databases, 4,652 of which were duplicates that were removed, leaving a sample of 1,664 studies. After screening by title and abstract, 1,590 studies were removed. The remaining studies were then screened against the inclusion criteria, resulting in inclusion of 18 studies in the qualitative analysis (Figure 1).

### Characteristics of included studies

Eighteen studies were included, 15 from the United States, one from the United Kingdom, one from Australia, and one from Israel. The total population was 29,750 participants, 9,043 of whom were recreational cannabis users. All participants were over the age of 18 and 14,180 were male. Table 2 presents the characteristics of the included studies.

**Table 2:** Characteristics of included studies (n = 18)

Author	Year	Country	Type of study	Instrument	Sample (n)	Cannabis users (n)
Diep et al.^11^	2021	United States	Cross-sectional	Questionnaire	21,729	3,132
Drazdowski et al.^12^	2021	United States	Cross-sectional	PSQI	354	354
McPherson et al.^13^	2021	United States	Cohort	PSQI	340	170
Skobic et al.^14^	2021	United States	Longitudinal	Questionnaire	1639	80
Wheeler et al.^15^	2021	United States	Cross-sectional	STMSHQ	79	20
Winiger et al.^16^	2021	United States	Cross-sectional	PSQI	152	152
Winiger et al.^17^	2020	United States	Cross-sectional	Questionnaire	1,882	1,882
Winiger et al.^18^	2019	United States	Cross-sectional	JHQ	1,656	1,656
Winiger et al.^19^	2021	United States	Cross-sectional	JHQ	760	760
Sznitman et al.^20^	2020	Israel	Cohort	Questionnaire	54	54
Altman et al.^21^	2019	United States	Cross-sectional	PSQI	311	311
Bolla et al.^22^	2008	United States	Cross-sectional	Polysomnography	31	17
Fisk et al.^23^	2007	United Kingdom	Cross-sectional	ESS and KSS	227	53
Karacan et al.^24^	1976	United States	Cross-sectional	EEG-EOG	64	32
Lim et al.^25^	2018	United States	Cross-sectional	SATED	107	107
Ly et al.^26^	2013	United States	Cross-sectional	PSQI	76	29
Maple et al.^27^	2016	United States	Cross-sectional	PSQI	41	41
Ogeil et al.^28^	2015	Australia	Cross-sectional	PSQI and ESS	248	85

EEG-EOG = Electroencephalography-electrooculography.

ESS = Epworth Sleepiness Scale; JHQ = Jessor Health Questionnaire; KSS = Karolinska Sleepiness Scale; PSQI = Pittsburgh Sleep Quality Index; STMSHQ = Saint Mary's Hospital Sleep Questionnaire.

The selected studies used six different scales to assess the sleep quality of their sample: the Pittsburgh Sleep Quality Index Questionnaire (PSQI), the Saint Mary's Hospital Sleep Questionnaire (SMHQ), the Jessor Health Questionnaire (JHQ), the Epworth Sleepiness Scale (ESS), the Karolinska Sleepiness Scale (KSS), and the Satisfaction Alertness Timing Efficiency Duration (SATED). The PSQI assesses sleep quality and changes, consisting of 19 questions that assess seven aspects (subjective sleep quality, sleep latency, sleep duration, habitual sleep efficiency, sleep changes, use of sleeping pills, and daytime sleep dysfunction) and the sum of the scores of the seven aspects ranges from 0 to 21 points – higher scores indicate greater change in sleep quality – and scores above 5 points already indicate dysfunction in the components of sleep.^29^

The SMHQ is a questionnaire consisting of 14 items that assess the quality of the previous night's sleep with questions that measure sleep duration, number of awakenings, and sleep satisfaction.^30^ The JHQ has two questions related to sleep: "How many hours do you typically sleep during the week?" and "How many hours do you typically sleep during the weekend?," designed to characterize sleep as short or long based on its duration.^19^ The ESS consists of eight items that assess the chance the patient would doze off or fall asleep in everyday situations, such as watching television or when stopped in traffic in the car, its score ranges from 0 to 24 points and the higher the score, the greater the level of sleep abnormality – with 10 points or more indicating that medical help is needed.^31^

The KSS measures an individual's sleepiness at a given time during the day, being indicated to measure sleep during work, jet lag, or situations that require attention, such as driving tests.^32^ The SATED scale measures some sleep-related characteristics, such as satisfaction, alertness, time, efficiency, and duration – a score of 10 indicates healthy sleep.^33^

### Methodological quality of the selected studies

The NOS scale was used to assess the quality of each study based on three domains: selection, comparability, and outcome. The articles are evaluated with a star rating, ranging from zero to nine stars and the higher the number of stars, the better the quality of the article. In this study, we chose to represent the scale in absolute numbers. As shown in Table 1, the studies rated as having excellent methodological quality were Wheeler et al.^15^ and Winiger et al.,^17^ both with a total score of 9. The studies with the lowest score, 6 points, were Skobic et al.^14^ and Lim et al.,^25^ which lost most points in the sample representativeness domain. The studies by Bolla et al.^22^ and Karacan et al.^24^ achieved maximum scores for outcome, assessing sleep by polysomnography and by electroencephalogram-electrooculogram respectively, whereas the other studies relied on self-report to conduct their assessments.

The JBI assessment rated studies by Wheeler et al.,^15^ Winiger et al.,^16,17,19^ Altman et al.,^21^ Bolla et al.,^22^ Fisk et al.,^23^ Lim et al.,^25^ Ly et al.,^26^ Maple et al.,^27^ and Ogeil et al.^28^ as high quality. The Skobic et al.^14^ study had the lowest quality score, since it did not use three of the evaluation items. The complete assessment list is shown in Table 3.

**Table 3:** Risk of bias in the included studies, according to the JBI critical appraisal checklist for analytical cross-sectional studies

Author	Were the criteria for inclusion in the sample clearly defined?	Were the study subjects and the setting described in detail?	Was the exposure measured in a valid and reliable way?	Were objective, standard criteria used for measurement of the condition?	Were confounding factors identified?	Were strategies to deal with confounding factors stated?	Were the outcomes measured in a valid and reliable way?	Was appropriate statistical analysis used?
Diep et al.^11^	No	Unclear	Yes	Yes	Yes	Yes	Yes	Yes
Drazdowski et al.^12^	No	No	Yes	Yes	Yes	Yes	Yes	Yes
McPhersonet al.^13^	Yes	No	Yes	Yes	Yes	Yes	Yes	Yes
Skobic et al.^14^	Yes	No	Yes	Yes	No	No	Yes	Yes
Wheeler et al.^15^	Yes	Yes	Yes	Yes	Yes	Yes	Yes	Sim
Winiger et al.^16^	Yes	Yes	Yes	Yes	Yes	Yes	Yes	Yes
Winiger et al.^17^	Yes	Yes	Yes	Yes	Yes	Yes	Yes	Yes
Winiger et al.^18^	No	No	Yes	Yes	Yes	Yes	Yes	Yes
Winiger et al.^19^	Yes	Yes	Yes	Yes	Yes	Yes	Yes	Yes
Sznitman et al.^20^	No	No	Yes	Yes	Yes	Yes	Yes	Yes
Altman et al.^21^	Yes	Yes	Yes	Yes	Yes	Yes	Yes	Yes
Bolla et al.^22^	Yes	Yes	Yes	Yes	Yes	Yes	Yes	Yes
Fisk et al.^23^	Yes	Yes	Yes	Yes	Yes	Yes	Yes	Yes
Karacan et al.^24^	Yes	Yes	Yes	Yes	Yes	No	Yes	Yes
Lim et al.^25^	Yes	Yes	Yes	Yes	Yes	Yes	Yes	Yes
Ly et al.^26^	Yes	Yes	Yes	Yes	Yes	Yes	Yes	Yes
Maple et al.^27^	Yes	Yes	Yes	Yes	Yes	Yes	Yes	Yes
Ogeil et al.^28^	Yes	Yes	Yes	Yes	Yes	Yes	Yes	Yes

## Discussion

The findings of this review demonstrate that cannabis use interferes with sleep in a predominantly negative way. Although participants in some studies had expected that marijuana could help with sleep quality, this effect was not achieved in most studies. This expectation was related to greater use of the substance by the participants and impaired sleep, especially among women.

Among the eighteen studies analyzed, 15 studies^11-14,16-19,22-28^ demonstrated that the use of marijuana has harmful effects on the sleep quality of users. However, the study by Sznitman et al.,^20^ observed a positive effect on one of the items evaluated, sleep latency, which is a measure of the time between transition from the waking state to full sleep and was decreased by marijuana use. The study by Altman et al.^21^ observed both negative and positive effects of recreational marijuana use on sleep quality. These findings may reflect the low monthly use of cannabis by the participants in the study by Altman et al.^21^ and the lack of use of validated measures by Sznitman et al.,^20^ such as the PSQI, the ESS, and/or polysomnography, compounded by a failure to control for confounding factors, such as use of sleeping pills or use of other substances that can interfere with sleep.

Of the sleep domains evaluated by the studies, sleep duration was the most affected by cannabis use. Diep et al.^11^ observed that heavy marijuana users were at greater risk of exhibiting extremes of sleep duration, i.e., sleeping for less than 6 hours or more than 9 hours, while recent marijuana users mainly had increased sleep duration. A slight increase was also observed in two other studies.^21,24^ Other studies predominantly showed decreased sleep duration. These findings were found to be associated with the ingestion of cannabis,^16^ early onset of cannabis use,^18^ and use of cannabis concomitantly with cocaine,^15^ demonstrating that cannabis can intensify the decrease in sleep duration seen in cocaine users. It is believed that early cannabis use can interfere with neural development, which can affect sleep in the future. Users with early onset of cannabis use have structural and functional changes in the prefrontal cortex and amygdala that can lead to changes in neuronal connections leading to sleep deprivation, altered sleep pattern, and poor sleep quality as they age.^13,18^

Other sleep aspects affected by cannabis use were sleep efficiency and daytime dysfunction. Sleep efficiency is the time the participant actually slept during the night of sleep and four studies^11,12,19,22^ found that it was decreased among marijuana users. Diep et al.^11^ observed this effect mainly in recent users, while Winiger et al.^19^ observed this finding in edible marijuana users. Two studies^12,28^ found an increase in daytime dysfunction among users and observed that this domain is related to greater problems with marijuana use. As a result, there is a subsequent worsening of sleep quality and an increase in marijuana consumption. These factors can be associated with the general impairment of sleep quality, which can cause irritability, lack of energy, negative impact on affection, decreased attention, impaired cognition, and worsening of memory and mood.^15,28^

The domain with the most divergent findings was sleep latency. In studies by Altman et al.^21^ and Sznitman et al.^20^ that used self-report questionnaires, users reported taking less time to fall asleep. In contrast, studies by Diep et al.,^11^ Bolla et al.,^22^ and Karacan et al.^24^ reported that marijuana users take longer to fall asleep, with the last two studies making observations using objective criteria, such as polysomnography and electroencephalography-electrooculography (EEG-EOG). Such discrepancies may occur, in part, because recreational marijuana formulations may vary. Part of the negative effects of cannabis on sleep quality is caused by its constituent delta-9-tetrahydrocannabinol (THC), which has a stimulant and hallucinogenic effect and can precipitate sleep disruption.^11^ On the other hand, cannabidiol (CBD), another constituent of marijuana, in medium to high doses has a sedative effect and is associated with improved sleep quality according to the PSQI, with increased sleep duration and improved sleep efficiency. In general, recreational forms of marijuana have a higher proportion of THC than CBD, which can impair sleep, although there are cannabinoid formulations with differences to this proportion, having less THC and more CBD than usual. These formulations are mainly found in medicinal use of the drug, but they can help to elucidate possible discrepancies in findings.^19^ Another possible explanation for these divergences is the low use of marijuana by participants in the study by Altman et al.^21^ and the lack of use of validated means of assessing sleep quality by Sznitman et al.^20^

The negative effects of cannabis on sleep quality were more often described among women.^12,13,26,28^ This finding can be explained by differences in the metabolism of substances among women, who may become dependent more quickly due to different metabolism and hormonal interactions with the drug^26^; this finding may be even more evident for women who started using marijuana at the beginning of adolescence.^13^ Moreover, women are at greater risk of having worse sleep quality due to social and cultural stressors,^12^ which explains the worse sleep quality in this group compared to men.

Several studies also showed that participants believed cannabis could improve their sleep problems,^12,14-16,21,22^ although its use does not seem to have had the desired effect of improving sleep quality.^12^ This finding is evident in men, users of other drugs, and unemployed people, who may use marijuana for this purpose more often than other evidence-based treatments, such as hypnotics or cognitive behavioral therapy, among others.^14^ The two biggest concerns regarding these findings relate to the influence these expectations have on increase in cannabis use^16,21,22^ and the risk of worsening sleep symptoms among users.^14^ Despite insomnia being one of the most cited reasons for using cannabis, the evidence base is not consistent to support its use recreationally in the general population, even in medicinal formulations. Indications are restricted to improving sleep quality in people with obstructive sleep apnea, post-traumatic stress disorder, and chronic pain.^11^

This systematic review showed that recreational use of marijuana interferes with users’ sleep, an effect observed both with subjective instruments, such as the PSQI, and with objective ones, such as polysomnography. Overall, most studies demonstrated a harmful effect of recreational cannabis on several domains of sleep quality. The limitations of this study include the non-inclusion of studies that evaluated the use of medical cannabis, which could have enabled identification of possible changes in sleep quality not described in this review. Furthermore, the cross-sectional nature of most of the articles does not allow long-term follow-up of the progressive effects that the continuous use of marijuana can have on sleep quality. Nevertheless, this study is important because it demonstrates that cannabis can have a harmful effect on sleep, since the mentality of cannabis users is that use of the drug contributes to improve sleep quality, an effect that, in general, was not observed by most of the studies.
